# Lack of Association of *LPA* Gene Polymorphisms with Coronary Artery Disease in Pakistani Subjects

**DOI:** 10.1155/2021/6692273

**Published:** 2021-06-11

**Authors:** Saleem Ullah Shahid, Shabana N A, Steve Humphries

**Affiliations:** ^1^Institute of Microbiology and Molecular Genetics, University of the Punjab, Lahore, Pakistan; ^2^Institute of Cardiovascular Sciences, University College London, UK

## Abstract

Coronary artery disease (CAD) is the leading cause of death worldwide. Pakistan faces a high epidemic of CAD, and the disease burden is increasing with the passage of time. Several genetic markers have been reported to be significantly associated with CAD; one of them is the lipoprotein A gene. The aim of the current investigation was to genotype the LPA gene SNPs, rs3798220 and rs10455872, in Pakistani subjects with CAD in a case control study design. The genotyping was done by TaqMan allelic discrimination assay. The results showed that the cases had significantly higher prevalence of diabetes (64.6%), hypertension (62.1%), and smoking habits (29.5%). The level of cholesterol in cases was higher than in controls (208.25 ± 54.11 vs. 175.34 ± 43.51, *p* ≤ 0.0001). The LDL-C was higher in cases than in controls (104.62 ± 37.94 vs. 77.05 ± 21.17, *p* ≤ 0.0001). Similarly, triglycerides were also higher in cases than in controls (214.51 ± 74.60 vs. 190.54 ± 70.26, *p* ≤ 0.0001), whereas HDL-C was lower in cases than in controls (45.13 ± 11.63 vs. 67.9 ± 17.57, *p* ≤ 0.0001). For rs3798220, the risk allele (C) frequency was 0.005 in cases and 0.002 in controls. For rs10455872, the risk allele (G) frequency was 0.017 in cases and 0.014 in controls. The risk allele frequencies were not significantly different between cases and controls (*p* > 0.05). In conclusion, these two *LPA* SNPs do not contribute significantly to CAD progression and cannot be used as independent risk factors for CAD in Pakistani population.

## 1. Introduction

Coronary artery disease (CAD) is a complex polygenic disease affecting individuals globally. A number of risk factors (modifiable and nonmodifiable) predispose to CAD [1]. Dietary habits, physical activity levels, genetic predisposition, and general health status interact to affect the outcome. In the recent years, many salivary mediators (e.g., galectin-3, interleukin-6, and urokinase-type plasminogen activator receptor) of CAD have been identified that clearly demonstrate the role of gingival health and other salivary components in the endothelial function [2–4].

Lipoprotein(a), LP(a), is a complex molecule consisting of an apolipoprotein, apo(a) attached to LDL molecule. The apo(a) of LP(a) is bound to LDL moiety at apolipoprotein B-100 (apoB-100) through a disulfide linkage [5]. The apo(a) is synthesized in the liver and then assembles with apoB-100 of LDL [6]. LP(a) inhibits plasminogen activation promoting thrombus formation [7]. It participates in the development of CAD through complex proatherogenic and prothrombotic pathways [8]. By accumulating in subendothelium, LP(a) accelerates cholesterol deposition, increased production of adhesion compounds, and aggregation of monocytes [9]. It can also bind to oxidized phospholipids mediating inflammation [10]. LP(a) thus can promote thrombosis either directly or by attenuating the thrombolysis.

The *LPA* gene (OMIM number 152200) is considered to be evolved from the plasminogen gene. The plasminogen gene with its 5 types of Kringle (K) domains (KI-KV) and a protease-like domain was first duplicated and then modified extensively to give rise the human *LPA* gene. During this remodelling process, the KI, KII, and KIII domains were lost while KV has retained a single copy. The protease-like domain changed and reshuffled so critically that it lost plasmin activity [11–13]. In the *LPA* gene, the KIV has been duplicated to KIV 1-10 and its copy number ranges from 2 to more than 40. This KIV-2 copy number polymorphism is responsible for variations in circulatory levels of LP(a) [14]. The LP(a) concentrations also vary among healthy people of different ancestries [15], and SNPs in the *LPA* gene explain much about the variability in the LP(a) concentration [16, 17].

*LPA* rs3798220 and rs10455872 are extensively studied SNPs in this gene; however, their association with CAD vanished when adjusted for LP(a) levels [8]. These SNPs are present in one of the haplotype blocks of the gene located towards the 5′ end. The nonsynonymous missense polymorphism rs3798220 substitutes methionine in place of isoleucine at position 4399 (Ile4399Me) and causes an increase in LP(a) concentration [10, 18, 19]. The rs10455872 is an intronic variant, 49 kb apart from rs3798220, and both are not in LD with each other. These SNPs may not be directly causal but tagging KIV-2 repeat polymorphism elsewhere in the gene, hence leading to protein size polymorphism [20]. Pakistani population represents a unique ethnic group with distinct genetic architecture [21]. Genetic markers identified in other population need to be validated in this ethnic group before establishing any management and treatment modality based on genetic factors. We, therefore, in the current study, investigated the two LPA SNPs in Pakistani CAD cases and controls to check their association with CAD.

## 2. Materials and Methods

The study comprised of 405 diagnosed CAD cases and 220 healthy controls. The criteria for the selection of study subjects have been described previously [1, 22–25]. The CAD cases were recruited from tertiary care hospitals in Lahore during February 2012 to June 2013. The selected subjects had suffered from a nonfatal myocardial infarction with the diagnosis made by the consultant cardiologists (from various hospitals including Ittefaq Hospital Trust, Jinnah Hospital, Mayo Hospital, and Doctors hospital) based on the reports of ECG, cardiac echo, angiography, troponine T/I, and clinical history. The inclusion criteria for CAD cases were that the subjects were recently diagnosed and had not started lipid-lowering or antihypertensive drug therapy. The inclusion criteria for controls were that the subjects were apparently healthy, not having any history of early CAD in the family. The cases and controls represented all socioeconomic groups. Subjects with obesity (BMI > 26 kg/m^2^ for Asian populations as described previously [26]) were excluded from the study to reduce the possible confounders but not those with type 2 diabetes because the number of CAD subjects with type 2 diabetes was high and the sample size would have become too small to have adequate power. Subjects seropositive for infectious diseases (HBV, HCV, and HIV) were excluded. All participants gave a written informed consent. The study was approved by the Ethics Committee, University of the Punjab, Lahore, and all procedures were in compliance with the Helsinki declaration.

### 2.1. Blood Sampling and Biochemical Analyses

Blood samples were taken after 8-12 hr fasting, and half sample was used for DNA isolation, while the rest half was used to obtain serum. Serum was separated by centrifuging gel vacutainers at 10,000 rpm for 10 min, collected in sterilized Eppendorf, and screened for any infectious agents (HBV, HCV, and HIV). Any positive samples were discarded, and safe samples were used for lipid profile determination. Serum total cholesterol (TC), triglycerides (TG), high-density lipoprotein cholesterol (HDL-c), and low-density lipoprotein cholesterol (LDL-c) were measured using commercially available kits (Spectrum Diagnostics, Egypt). Epoch BioTek microplate reader (BioTek Instruments, Highland Park) was used for all optical density measurements.

### 2.2. Genotyping

The DNA was extracted from whole blood leucocytes using Wizard genomic DNA purification Kit (Promega, USA). The DNA samples were quantified using nanodrop (ND-8000, USA). The concentration of DNA samples was standardized to 1.25 ng/*μ*l. The genotyping was carried out in specially designed 384-well plates (Micro Amp). The DNA samples were arrayed into plates by an automated robotic liquid handling system (Biomerk-FX, Beckman Coulter). The quality check of genotyping techniques was maintained by the inclusion of nontemplate controls (NTCs). There were 16 NTCs in each plate of 384 wells. Only those runs were included in the analysis where none of the NTCs crossed the amplification cutoff line. Only the samples which were clearly clustered towards their respective axes were included in the subsequent assay. Both the SNPs were genotyped by TaqMan technique. Because the risk alleles of both SNPs were very low in frequency in our samples, the genotypes were also randomly confirmed by direct DNA sequencing.

### 2.3. Statistical Analysis

The results were analyzed using Statistical Package for Social Sciences (SPSS) IBM, version 22. Continuous variables like blood lipid levels were compared between cases and controls using an independent sample Student *t-*test. Hardy Weinberg equilibrium was assessed by a *χ*^2^ goodness of fit test. For low-risk allele frequency, we used Fisher's exact test for comparing allele frequencies between cases and controls rather than using a chi-square test. Since CAD is a binary variable, the association of the SNPs with CAD was examined using binary logistic regression. The CAD status was considered a dependent variable for different analyses.

## 3. Results

### 3.1. Study Subjects' Characteristics

The baseline biochemical and anthropometric characteristics of the subjects under study are given in Table 1. The cases were more diabetic and hypertensive; smoking rate was also higher in cases than controls. Total cholesterol (TC), triglycerides (TG), and LDL-C were significantly higher, whereas HDL-C was lower in cases than controls. Body mass index (BMI) did not differ significantly between the cases and controls.

### 3.2. LPA Variants and CAD

The features of the SNPs under study are given in Supplementary Table 1. Both SNPs gave good call rates and neither differed significantly from Hardy Weinberg equilibrium. The genotyping results showing a clear separation between the common homozygotes and heterozygotes of rs3798220 are shown (supplementary Figures 1 and 2). There was not even a single homozygous for the risk allele observed for this SNP in the subjects under study. For the SNP rs10455872, there was 1 homozygous form of the risk allele and distinct clusters were present for common homozygotes and heterozygotes. The direct DNA sequencing results for common homozygotes and risk heterozygotes for both SNPs are shown in supplementary figures.

For rs3798220, the risk allele (C) frequency was 0.005 in cases and 0.002 in controls. While for rs10455872, the risk allele (G) frequency was 0.017 in cases and 0.014 in controls. The risk allele frequencies were not significantly different between cases and controls as shown by Fisher's exact test *p* value (Table 2). The SNPs were not associated with CAD in the studied subjects as shown by their CAD OR *p* values (Table 3).

## 4. Discussion

CAD is the leading cause of death globally despite the measures being taken. The management of heart diseases poses a huge health care burden. Therefore, if the CAD lifetime risk of an individual can be calculated either using genetic markers or serum biomarkers, this can lead to significant reduction of health care burden. The risk score analysis based on genetic markers is routinely done in the developed countries along with Framingham risk score, and some preliminary data is also available for Pakistani [1, 27]. The use of serum biomarkers (left ventricular ejection fraction determination) has also proved useful; however, in most of the cases, the symptoms begin after the disease has already progressed to an advanced stage [25, 28].

In this study, the association of two *LPA* gene polymorphisms, rs3798220 and rs10455872, with CAD has been studied in Pakistani population. The risk alleles were very low in frequency and were not significantly high in cases than in controls. Due to very low-risk allele frequencies in the target population, these SNPs were not adequately powered to establish an association with CAD in Pakistani population.

The association of *LPA* polymorphisms with CAD varies among ethnicities due to the difference in allele frequencies among different populations. In our studied subjects, the risk allele frequency of *LPA* rs3798220 was very low (0.04%) due to which the study was not powered to detect a significant increase in outcome despite a 2.18-fold increase in the odds of CAD. However, the association of this SNP with CAD has been reported in Caucasians [10, 18]. The low-risk allele frequency (<1%) of this SNP suggests that it accounts for only minor variations in CAD incidence. Contrary to this, [29] in another study on Brazilian population, the risk allele frequency was pretty high (6.2%) but this study failed to find the association of the SNP with CAD, whereas, in a prospective study on healthy people, the risk allele was 3.7% and increased CAD events 2 times [30].

The SNP *LPA* rs10455872 had low RAF (0.16%) and was not associated with CAD risk. However, in a Brazilian study, the risk allele frequency was 6.4% and the SNP was significantly associated with CAD [29]. So far, in the studies carried out in European Caucasians, the association of *LPA* polymorphisms with CAD has been established [20], but the association of *LPA* rs3798220 with CAD was not replicated in a study on Chinese [5]. In another study comparing European Caucasian, South Asians, and Chinese subjects regarding the genetic polymorphisms of *LPA*, it was found that *LPA* rs10455872 was only prevalent in Caucasians [15].

## 5. Conclusion

In conclusion, the low-risk allele frequency of the LPA SNPs is an indication that these SNPs have recently emerged and are not evolutionary conserved. These SNPs are ethnicity specific and affect the disease in specific environmental conditions. According to our findings, these two *LPA* SNPs are not CAD associated and cannot be used as independent risk factors for CAD in Pakistani population. However, if other *LPA* polymorphisms or haplotypes are included, enough power may be obtained for genetic markers to be considered independent CAD risk factors.

## Figures and Tables

**Table 1:** Anthropometric and biochemical parameters of study subjects.

Variables	Cases	Controls	*p* value
Number	405	220	
Age (years)	59.1 ± 12.6	56 ± 10.5	0.002
Sex			
Males (*n*)	216	120	0.27
Females (*n*)	189	100	
BMI (kg/m^2^)	22.46 ± 6.75	21.46 ± 9.11	0.119
Diabetes (%)	64.6	13.6	5.1 × 10^−34^
Hypertension (%)	62.1	16.4	8.9 × 10^−28^
Smoking (%)	29.5	10.5	7.3 × 10^−08^
Total cholesterol	207.5 ± 53.7	175.4 ± 43	8.8 × 10^−14^
Triglycerides	212.4 ± 70	188 ± 66.3	2.6 × 10^−5^
LDL-C	106 ± 28.9	84.7 ± 17	6.3 × 10^−22^
HDL-C	45.2 ± 11.9	67.4 ± 16.3	1.8 × 10^−66^

**Table 2:** Comparison of RAFs between cases and controls.

SNP			RAFs			
	Alleles	Risk allele	Cases	Controls	^∗^ *p* value	Total
rs3798220	T/C	C	0.005	0.002	0.66	0.004
rs10455872	A/G	G	0.017	0.014	0.81	0.016

^∗^Fisher's exact test *p* value.

**Table 3:** CAD OR of the studied SNPs.

SNP	OR	95% CI	*p* value
rs3798220	2.2	0.24-19.63	0.49
rs10455872	1.25	0.49-3.16	0.64

## Data Availability

All data is available with the corresponding author and can be accessed on request.

## Supplementary Materials

Supplementary Materials Supplementary Table 1: basic features of SNPs under study. Supplementary Figure 1(a): genotyping results of rs3798220 for cases. Supplementary Figure 1(b): sequencing results of rs3798220. Supplementary Figure 2(a): genotyping results of rs10455872 for cases. Supplementary Figure 2(b): sequencing results of rs10455872.
