# Supplementary material for: Lack of Association of LPA Gene Polymorphisms with Coronary Artery Disease in Pakistani Subjects
Source: Dis Markers. 2021 Jun 11;2021:6692273. doi: 10.1155/2021/6692273 (PMC8214490; doi:10.1155/2021/6692273)
Supplement: Supplementary Materials — Supplementary Table 1: basic features of SNPs under study. Supplementary Figure 1(a): genotyping results of rs3798220 for cases. Supplementary Figure 1(b): sequencing results of rs3798220. Supplementary Figure 2(a): genotyping results of rs10455872 for cases. Supplementary Figure 2(b): sequencing results of rs10455872. [file 6692273.f1.docx]

**Supplementary Table 1. Basic features of SNPs under study**

| Gene | CHR | SNP | Call rate % | HWE-*p* | | |
| --- | --- | --- | --- | --- | --- | --- |
|  |  |  |  | Cases | Controls | Total |
| *LPA* | 6q25 | rs3798220 | 95 | 1.000 | 1.000 | 1.000 |
| *LPA* | 6q26 | rs10455872 | 96 | 0.100 | 1.000 | 0.145 |

HWE-*p* is Hardy Weinberg equilibrium Fisher’s exact


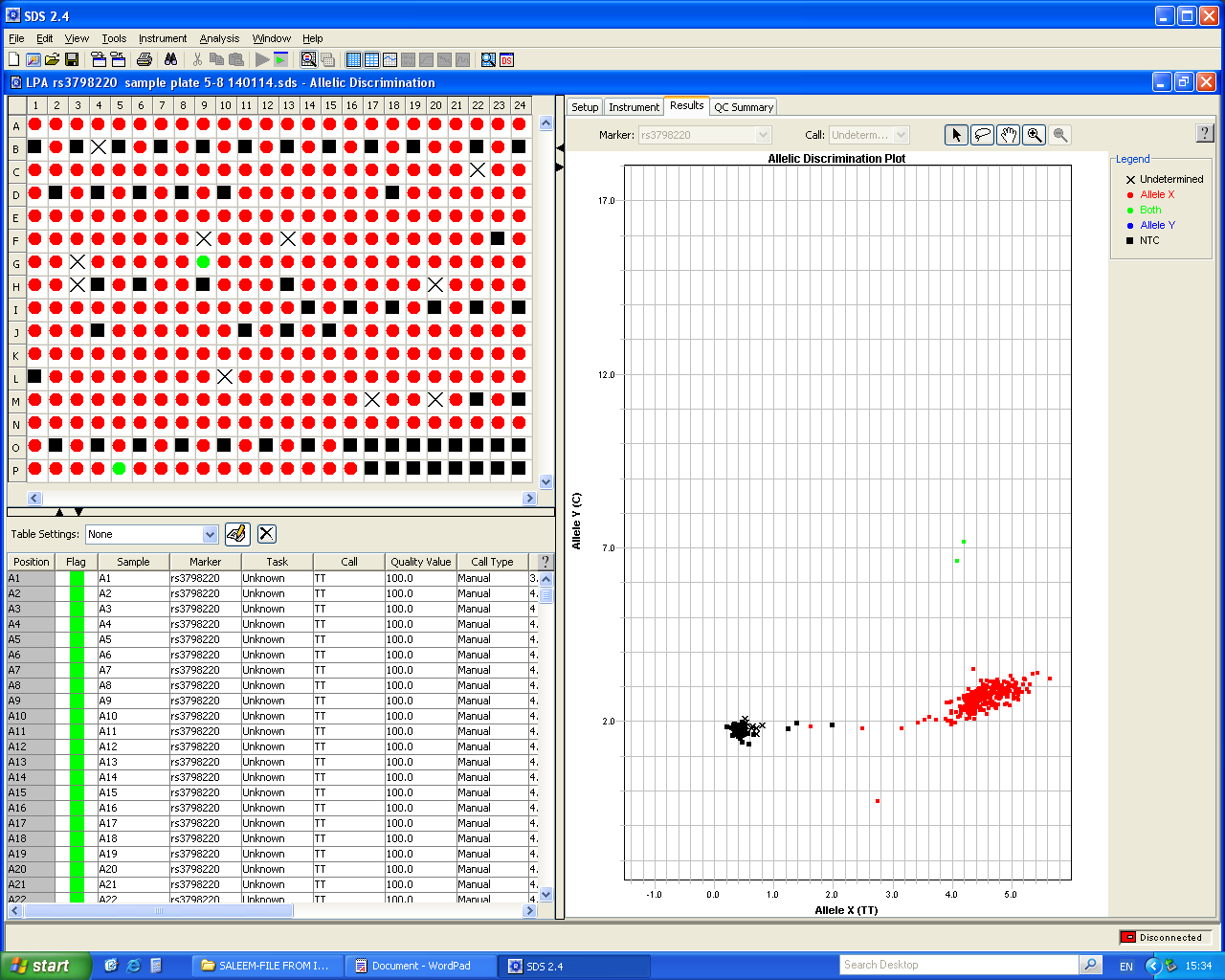


Supplementary Figure 1(a). Genotyping results of rs3798220 for cases


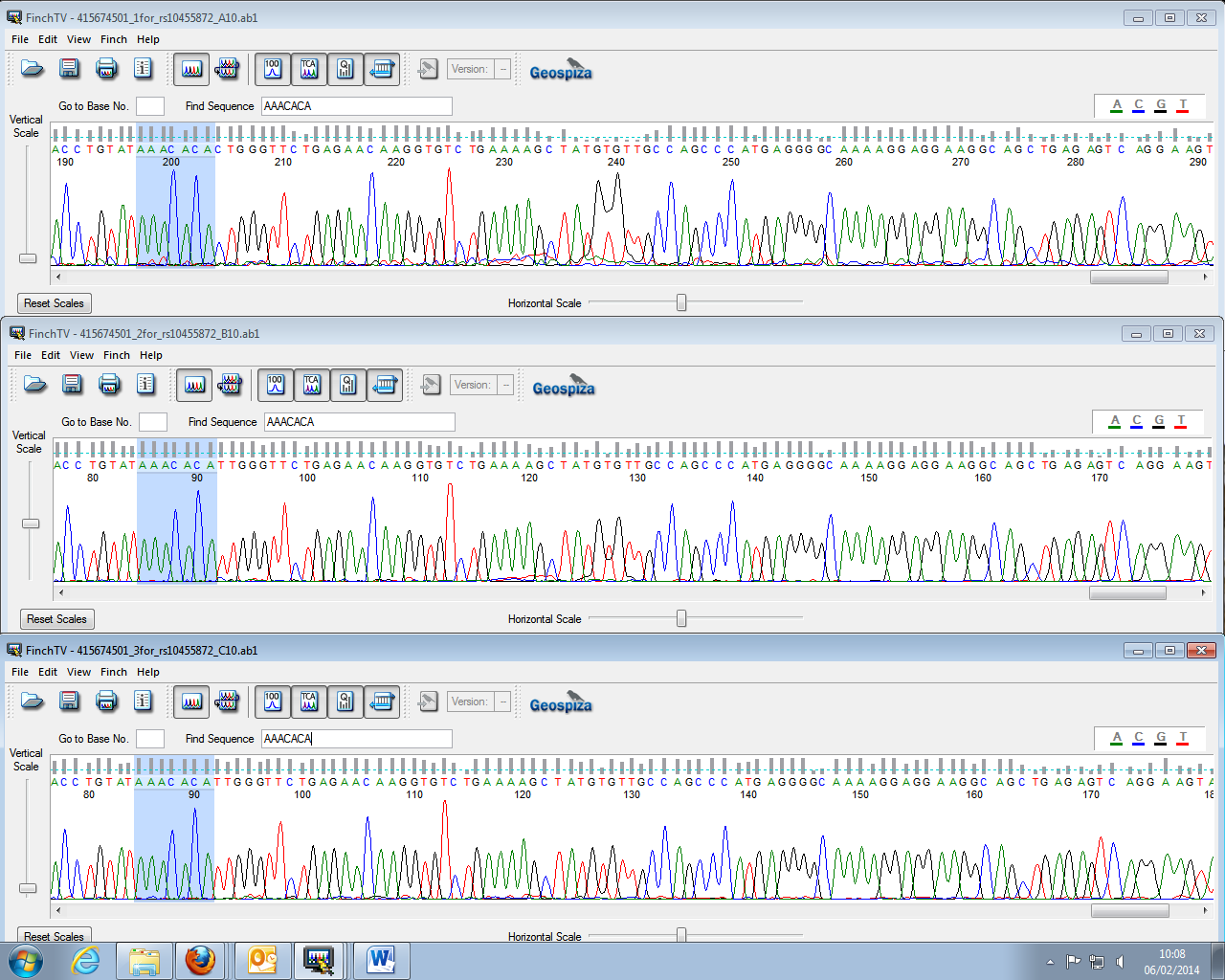


Supplementary Figure 1(b). Sequencing results of rs3798220. The upper half of the figure is a heterozygous individual for risk allele (C allele at the end of shaded area). Lower half is a common homozygous (T allele at the end of shaded area).


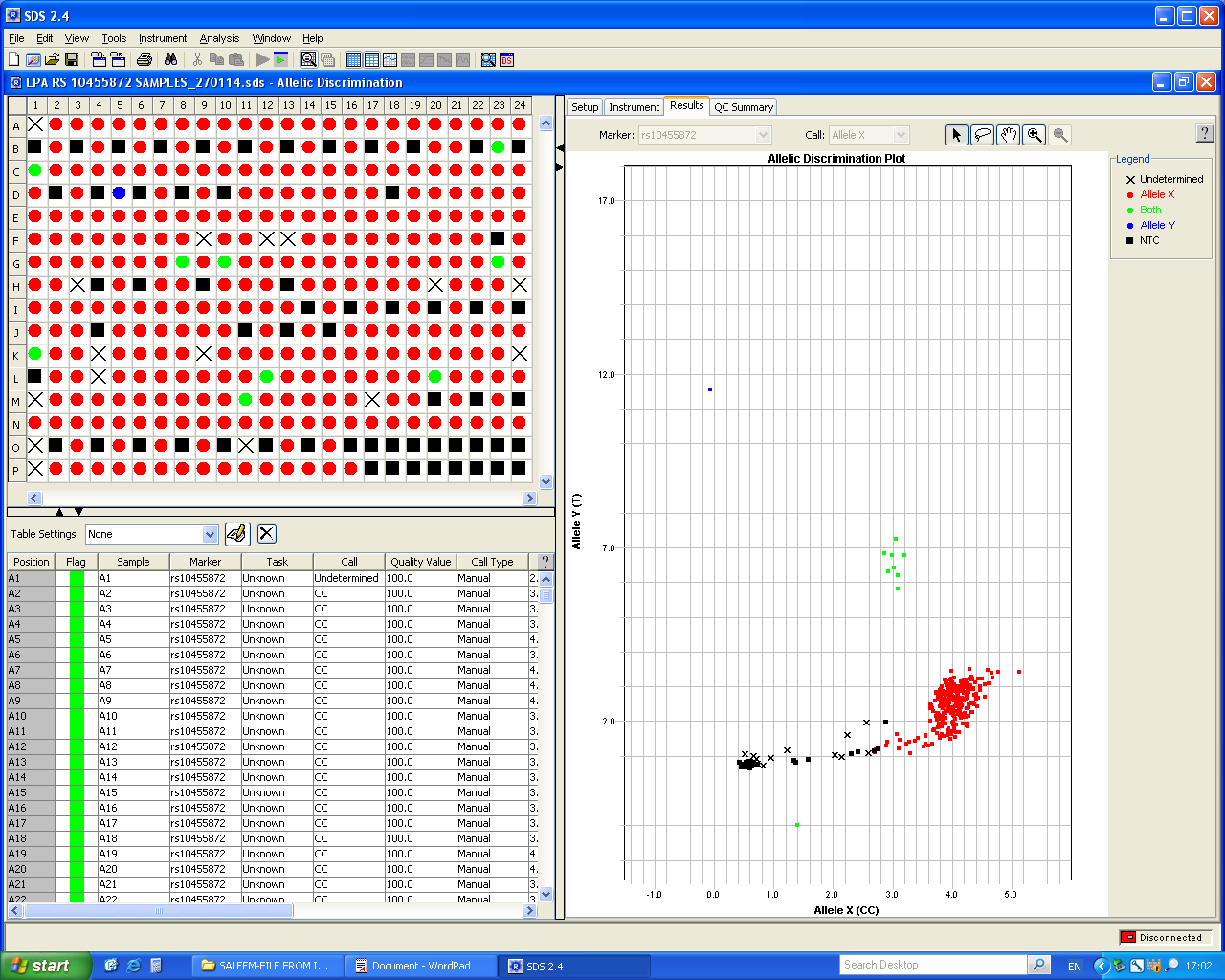


Supplementary Figure 2(a). Genotyping results of rs10455872 for cases


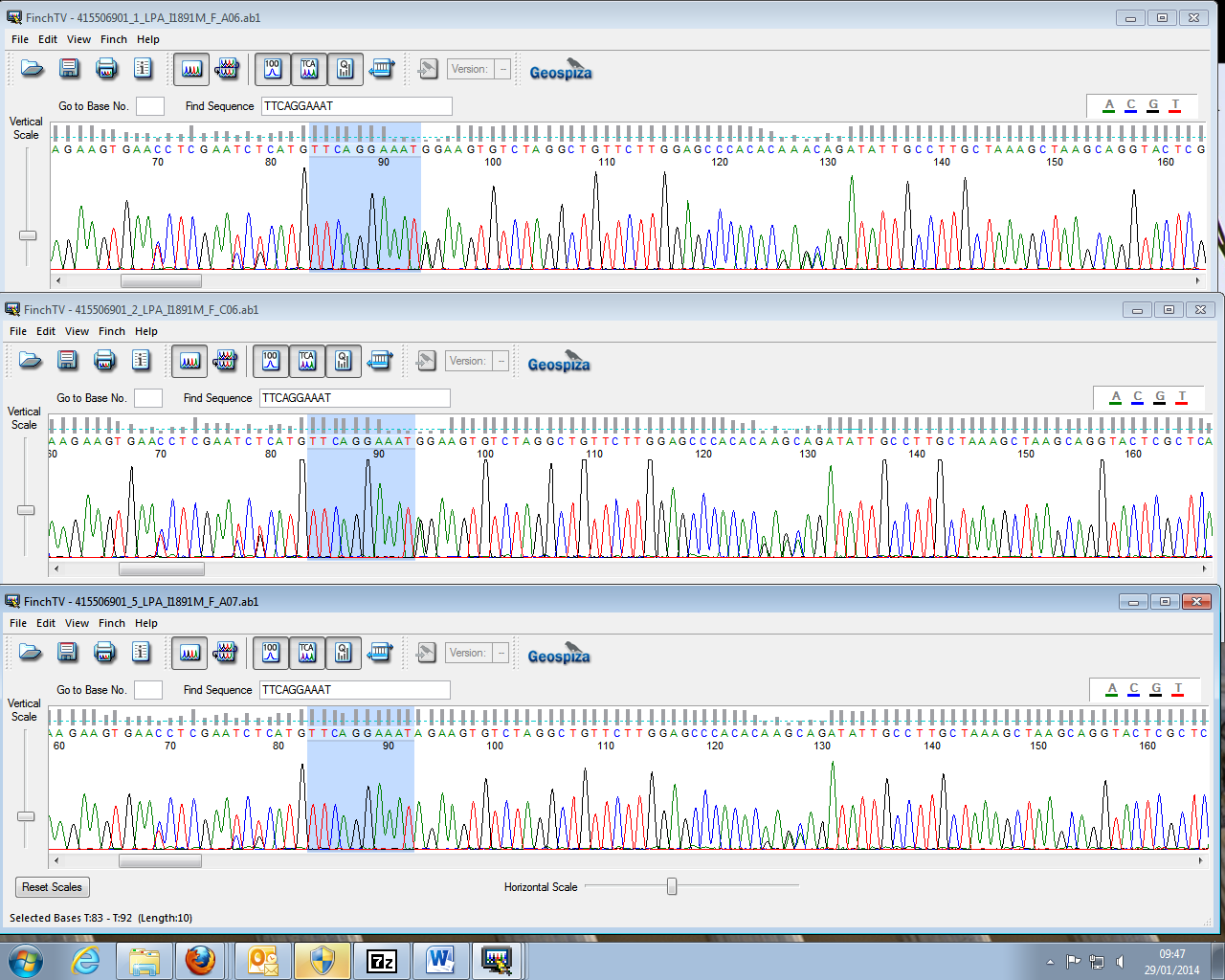


Supplementary Figure 2(b). Sequencing results of rs10455872. The upper half of the figure is a heterozygous individual for risk allele (G allele at the end of shaded area). Lower half is a common homozygous (A allele at the end of shaded area).
